# The FOCU.SE trial: a nationwide Swedish drug repurposing protocol and research framework

**DOI:** 10.2340/ao.v65.45355

**Published:** 2026-04-13

**Authors:** Edvard Abel, Päivi Östling, Ebba Hallersjö Hult, Katarzyna Kulbacka, Haris Babacic, Annika Baan, Ana Carneiro, Luigi De Petris, Henrik Fagman, Signe Friesland, Oskar Frisell, Mats Hellström, Gabriel Lindahl, Katarina Steen Carlsson, David Tamborero, Antonios Valachis, Daniel Öhlund, Janne Lehtiö, Richard Rosenquist, Anders Edsjö, On behalf of the FOCU.SE trial group including, Johan Botling, Helena Bäckvall, Rikard Fred, Stina Garvin, Andreas Hallqvist, Markus Heidenblad, Tina Catela-Ivkovic, Anita Koskela von Sydow, Linda Köhn, Rozina Caridha Lagerhorn, Maja Löfgren, Malin Melin, Martin Isaksson-Mettävainio, Lotte NJ Moens, Lars Ny, Helena Nord, Hannes Olauson, Alvida Qvick, Stefano Rapisarda, Emma Tham

**Affiliations:** a Department of Oncology, Institute of Clinical Sciences, University of Gothenburg, Gothenburg, Sweden; b Sahlgrenska Comprehensive Cancer Centre, Department of Oncology, Sahlgrenska University Hospital, Region Västra Götaland, Gothenburg, Sweden; c Science for Life Laboratory (SciLifeLab), Department of Oncology-Pathology Karolinska Institutet, Stockholm, Sweden; d Karolinska Comprehensive Cancer Project Unit, Karolinska University Hospital, Stockholm, Sweden; e Stockholm School of Economics Institute for Research, Stockholm, Sweden; f Department of Oncology and Pathology, Karolinska Institutet and SciLifeLab, Stockholm, Sweden; g Department of Oncology, Lund University Cancer Center, Institution för Clinical Sciences, Lunds University, Lund, Sweden; h Department of Hematology, Oncology and Radiation Physics, Skåne University Hospital Comprehensive Cancer Center, Skåne University Hospital, Lund, Sweden; i Center for Clinical Cancer Studies, Karolinska University Hospital, Stockholm, Sweden; j Department of Oncology and Pathology, Karolinska Institutet, Stockholm, Sweden; k Department of Laboratory Medicine, Institute of Biomedicine, Sahlgrenska Academy, University of Gothenburg, Gothenburg, Sweden; l Department of Clinical Pathology, Sahlgrenska University Hospital, Gothenburg, Sweden; m Department of Head-Neck-, Lung-Cancer and Melanoma, Theme Cancer, Karolinska University Hospital, Stockholm, Sweden; n The Swedish Institute for Health Economics (IHE), Lund, Sweden; o Department of Learning, Informatics, Management and Ethics (LIME), Karolinska Institutet, Stockholm, Sweden; p Department of Immunology, Genetics and Pathology, Uppsala University, Uppsala, Sweden; q Department of Oncology and Department of Biomedical and Clinical Sciences, Linköping University, Linköping Sweden; r Department of Clinical Sciences, Lund University, Malmö, Sweden; s Department of Research, Development, Education and Innovation, Skåne University Hospital, Lund, Sweden; t Division of Pathology, Karolinska University Hospital, Stockholm, Sweden; u Department of Oncology, Faculty of Medicine and Health, Örebro University, Örebro, Sweden; w Department of Diagnostics and Intervention, and Wallenberg Centre for Molecular Medicine, Umeå University, Umeå, Sweden; x Department of Molecular Medicine and Surgery, Karolinska Institutet, Stockholm, Sweden; y Clinical Genetics and Genomics, Karolinska University Hospital, Stockholm, Sweden; z Department of Clinical Genetics, Pathology and Molecular Diagnostics, Skåne University Hospital, Region Skåne, Lund, Sweden; aa Division of Pathology, Department of Clinical Sciences, Lund University, Lund, Sweden; bb Clinical Department of Clinical Pathology, Region Östergötland, Linköping, Sweden; cc Center for Translational Genomics, Lund University, Lund, Sweden; dd Clinical Genomics Lund, SciLifeLab, Lund, Sweden; ee Clinical Research Center, Faculty of Medicine and Health, Örebro University, Örebro, Sweden; ff Department of Medical Biosciences, Umeå University, Sweden; gg Department of Clinical Genetics and Genomics, Sahlgrenska University Hospital, Gothenburg, Sweden; hh Department of Immunology, Genetics and Pathology, Rudbeck Laboratory, Uppsala University, Uppsala, Sweden; ii Clinical Genomics Uppsala, Science for Life Laboratory, Uppsala University, Uppsala, Sweden; jj Department of Immunology, Genetics and Pathology, Uppsala, Science for Life Laboratory, Uppsala University, Uppsala, Sweden; kk Clinical Pathology, Uppsala University Hospital, Uppsala, Sweden; ll Department of Clinical Pathology and Cancer Diagnostics, Karolinska University Hospital

**Keywords:** Precision medicine, biomarker profiling, drug repurposing, targeted therapy, platform trial, infrastructure, multimodal analysis, Sweden, PRIME-ROSE consortium, cancer genomics, proteomics

## Introduction

Advances in genomic technologies have transformed cancer classification and treatment selection, enabling more personalized therapeutic strategies [1]. Despite this, many patients with advanced cancer do not benefit from genomic profiling due to regulatory barriers, limited treatment access, and insufficient clinical evidence supporting biomarker-guided therapies [2]. Drug repurposing, using approved drugs based on molecular alterations rather than tumour histology, offers a pragmatic solution, as demonstrated by trials such as Targeted Agent & Profiling Utilization Registry (TAPUR) (USA) and DRUP (Netherlands) [3, 4] Inspired by the Drug Rediscovery Protocol (DRUP), several national precision oncology trials have been launched across Europe, including initiatives in Denmark, Norway, and Finland [5–7].

In Sweden, the National Life Science Strategy prioritizes equitable access to precision medicine. The MEGALiT trial provided an initial national experience with a DRUP-like approach, while collaborations within Testbed Sweden Precision Health Cancer and European initiatives such as PCM4EU and PRIME-ROSE have paved the way toward a national effort [8, 9]. Building on this groundwork, the FOCU.SE trial was developed as a nationwide platform to support biomarker-driven cancer treatment and integrated data generation.

FOCU.SE leverages national infrastructure through Science for Life Laboratory (SciLifeLab) and Genomic Medicine Sweden (GMS). Beyond the clinical trial component (FOCU.SE-Trial), the platform includes FOCU.SE-Explore, which enables exploratory multi-omics analyses, including whole-genome sequencing (WGS), whole-transcriptome sequencing (WTS), and proteomics. The complementary FOCU.SE-Data framework aims to integrate clinical, molecular, and outcome data into a unified ecosystem to support continuous learning and innovation in precision cancer medicine.

## Patients/material and methods

### Study design

FOCU.SE is a nonrandomized, pragmatic, phase II exploratory platform trial evaluating biomarker-guided off-label use of European Medical Agency (EMA)-approved targeted therapies. The design combines basket and umbrella trial features, enabling evaluation of targeted treatments across cancer types and biomarkers [10]. Each cohort consists of patients sharing a specific cancer type, molecular alteration, and treatment. A nested biomarker study allows prospective collection of samples irrespective of trial inclusion for exploratory research.

### Population

All seven Swedish University Hospitals participate in patient recruitment. Eligible patients are adults (≥18 years) with histologically confirmed locally advanced or metastatic cancer, measurable disease, European Cooperative Oncology Group (ECOG) performance status 0–2, adequate organ function, and no remaining standard treatment options. Key exclusion criteria include rapidly progressive disease, significant cardiac dysfunction, pregnancy, or life expectancy under 6 months.

### Screening and biomarker profiling

Patients undergo comprehensive genomic profiling primarily using GMS-developed targeted sequencing panels for solid or hematologic malignancies, supplemented by other validated assays and, when tissue is unavailable, by circulating tumour DNA (ctDNA) analysis. The tissue profiling is performed at each including site as part of the routine diagnostic workflow and is funded by research grants. Analyses of ctDNA and exploratory analyses are centralized with equal access for all sites. The GMS560 panel covers over 500 genes and detects a wide range of genomic alterations, base substitutions (SNVs), insertions and deletions (INDELs), copy-number variations (CNVs), and gene rearrangements (fusions) as well as more complex biomarkers such as microsatellite instability (MSI) and tumour-mutational burden (TMB) [11]. All actionable findings are reviewed by a national Molecular Tumor Board (MTB) using the MTB Portal as a decision-support tool [12]. When multiple actionable alterations are present, treatment prioritization follows predefined criteria. Optional pre-screening is permitted earlier in the disease course.

### Exploratory biomarker study (FOCU.SE-Explore)

Tumour tissue and blood samples are systematically collected for exploratory analyses. Baseline proteomics is performed on all available cases, preferably using fresh-frozen tissue but also from Formalin-Fixed Paraffin-Embedded (FFPE) samples. Additional analyses, including WGS, WTS, tissue imaging, long-read sequencing, and spatial omics, are conducted based on treatment rationale and sample availability.

### Data integration

FOCU.SE-Data provides harmonized clinical data collection and aims to establish a national, data-driven ecosystem integrating clinical data, biobanking, diagnostics, molecular analyses, and outcomes. The framework supports future development of multimodal diagnostics, artificial intelligence (AI)-driven analytics, and knowledge bases, addressing technical and regulatory challenges in parallel with trial execution.

### Treatment assignment

Treatments are selected from a predefined catalog of EMA-approved targeted therapies. Four drugs (olaparib, Poly(ADP-ribose) polymerase [PARP]-inhibitor; tepotinib, targeting proto-oncogene c-MET (MET); amivantamab, targeting Epidermal Growth Factor Receptor (EGFR)/MET; ivosidenib, targeting Isocitrate Dehydrogenase 1 [IDH1]) have been included in the current protocol, but new therapeutical arms will be added as they become available. Initial Assignment is guided by the European Society of Medical oncology (ESMO) Scale for Clinical Actionability of Molecular Targets (ESCAT) [13]. Drugs are administered according to approved dosing, and both monotherapies and previously evaluated combinations are allowed.

### Cohort design

Each biomarker–drug–cancer cohort follows a Simon two-stage design with early stopping for futility [14]. In stage 1, eight patients are enrolled; if at least one patient demonstrates clinical benefit (objective response or stable disease at 16 weeks), the cohort proceeds to stage 2 with an additional 16 patients. If five or more patients demonstrate benefit in stage 2, further investigation is supported. Selected cohorts may expand to a stage 3 with up to 130 additional patients, subject to agreement with sponsors and drug providers.

### Statistical considerations

Analyses include descriptive statistics for efficacy and safety, Kaplan–Meier estimates for time-to-event outcomes, and exploratory health economic evaluations. Key outputs include quality-adjusted life years (QALYs), incremental cost-effectiveness ratios (ICERs), and total costs.

## Endpoints

*Primary endpoints* include disease control rate (Complete Response [CR]/Partial Response [PR]/ Stable Disease [SD] at 8 and 16 weeks), proportion of eligible patients accessing recommended therapies, and safety assessed by CTCAE v5.0.

*Secondary endpoints* include progression-free survival, overall survival, Health-related Quality of Life (measured by European Organisation for Research and Treatment of Cancer (EORTC) Quality of Life Questionnaire (QLQ)-C30, European Quality of Life Five Dimension Five Level Scale (EQ-5D-5L), and Hospital Anxiety and Depression Scale (HADS) questionnaires), and cost-effectiveness.

*Exploratory endpoints* assess feasibility and yield of multi-omics profiling, serial ctDNA dynamics, and development of predictive biomarkers.

## Discussion

FOCU.SE represents a major paradigm shift in the national implementation of precision cancer medicine (PCM) in Sweden by tightly linking biomarker-driven diagnostics, oncology care, and translational research within a single coordinated framework. By integrating molecular diagnostics, structured access to targeted treatments, and standardized data sharing, the platform establishes a sustainable model for real-world evaluation of targeted drug repurposing strategies and substantially lowers the threshold for explorative analyses across treatment cohorts.

Importantly, FOCU.SE reduces regional disparities in access to advanced diagnostics and targeted therapies by enabling that patients throughout Sweden are evaluated using harmonized workflows and evidence-based selection criteria. To monitor nationwide implementation, data on the distribution of included patients across Sweden will be reported to the National Board of Health and Welfare. The platform systematically generates real-world data on treatment effectiveness, safety, and health-economic outcomes, thereby providing robust data to inform reimbursement decisions, clinical guidelines, and regulatory expansion of targeted therapies. Like in other DRUP-like clinical trials, rare cancers may be overrepresented but more common malignancies will also be included, allowing for potential new indications also for larger patient groups.

**Figure 1:**
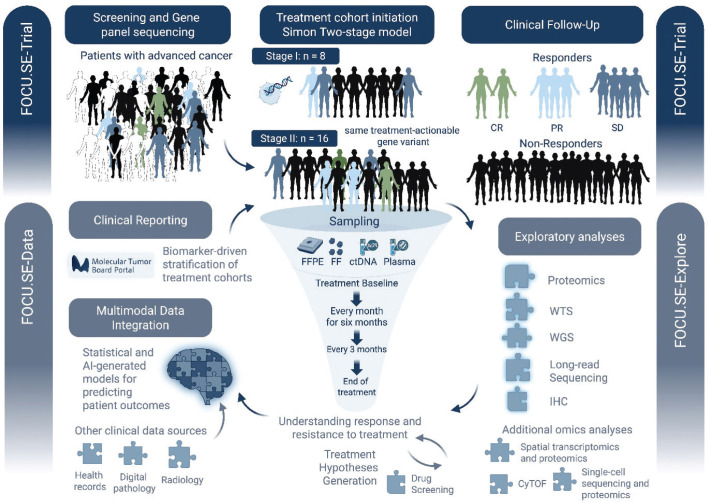
Schematic view of FOCU.SE platform.

The platform’s modular structure allows the continuous integration of new drugs and biomarkers within FOCU.SE-Trial and serves as a foundation for advanced multimodal omics profiling through FOCU.SE-Explore. Specifically, all molecularly characterized patients will undergo high-resolution proteomic profiling for assessing whether integration of proteomic and genomic data will improve clinical benefit and patient selection. These efforts aim to deepen the understanding of treatment response mechanisms, while FOCU.SE-Data supports integrated data analysis and secure data accessibility (Figure 1). The national MTB platform will also be able to identify patients for other biomarker-driven clinical trials beyond the present study.

**Figure 2:**
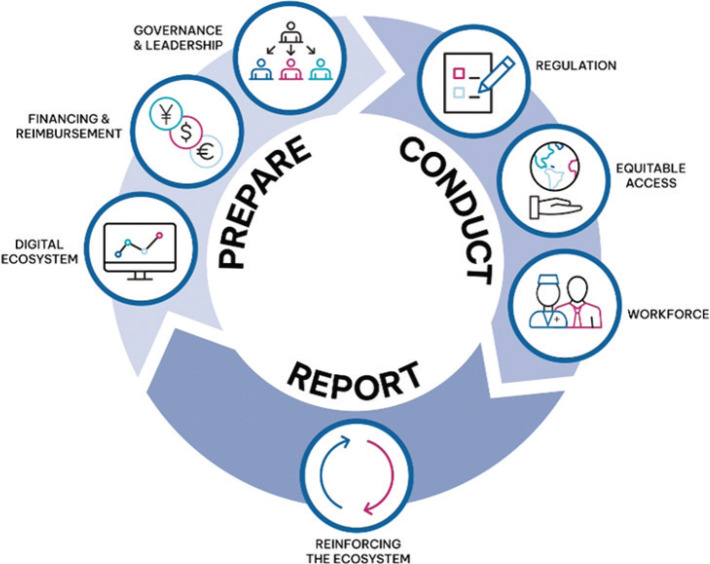
Structure of the implementation framework for PCM clinical trials.

FOCU.SE also exemplifies how clinical trials can function as testbeds for health system readiness in PCM (Figure 2). Beyond evaluating clinical outcomes, the trial addresses organizational, infrastructural, and data-related requirements necessary for large-scale PCM implementation within routine cancer care. By integrating advanced diagnostics, coordinated data flows, and multidisciplinary collaboration, FOCU.SE contributes to understanding how health systems can equitably and sustainably adopt personalized cancer care. Insights from the trial align with emerging implementation frameworks that emphasize early consideration of health system capacity, ensuring that innovations tested in research settings can be translated into real-world clinical practice [15].

Furthermore, as part of the European Horizon Cancer-funded PRIME-ROSE consortium, FOCU.SE contributes to validation of biomarker–drug outcome relationships and to the development of a European PCM infrastructure, strengthening interoperability and scientific exchange across national borders [9]. By harmonizing study design and endpoints to the other trials within PRIME-ROSE, FOCU.SE will add to the structured data sharing enabling aggregation of molecularly defined cohorts. This formalized collaboration between DRUP-like clinical trials has already established itself as a key interface for pharmaceutical companies and has enabled pooling of data from multiple patient cohorts, facilitating substantially faster evidence generation and earlier conclusions on drug effectiveness.

### Conclusion

The FOCU.SE trial platform provides a nationwide clinical infrastructure for the implementation of PCM in Sweden. By enabling biomarker-guided use of approved drugs outside of their current indication within a structured clinical trial framework and supporting systematic evaluation of clinical outcomes, the platform aims to accelerate equitable access to personalized cancer therapy across healthcare regions. In parallel, it generates high-quality real-world evidence to support regulatory decision-making and potential expansion of indications for targeted therapies.

## Data Availability

Data not available yet.
